# Implementation of Steiner Point of Fuzzy Set

**DOI:** 10.1155/2014/593065

**Published:** 2014-04-09

**Authors:** Jiuzhen Liang, Dejiang Wang

**Affiliations:** Department of Computer Science, Jiangnan University, Wuxi, Jiangsu 214122, China

## Abstract

This paper deals with the implementation of Steiner point of fuzzy set. Some definitions and properties of Steiner point are investigated and extended to fuzzy set. This paper focuses on establishing efficient methods to compute Steiner point of fuzzy set. Two strategies of computing Steiner point of fuzzy set are proposed. One is called linear combination of Steiner points computed by a series of crisp **α**-cut sets of the fuzzy set. The other is an approximate method, which is trying to find the optimal **α**-cut set approaching the fuzzy set. Stability analysis of Steiner point of fuzzy set is also studied. Some experiments on image processing are given, in which the two methods are applied for implementing Steiner point of fuzzy image, and both strategies show their own advantages in computing Steiner point of fuzzy set.

## 1. Introduction

Associated with every closed bounded convex set in ℝ^*n*^ is a point known as Steiner point or curvature centroid [1]. Though the Steiner point of smooth convex curves was defined and investigated by Seiner already in 1840, some properties, like additivity, were discovered almost in 1960s by Shephard [2]. From then on, Steiner point was known as one of the important geometry points and was studied more and more. Nowadays, the Steiner point is regarded as the only point-valued function defined for all convex bodies, which is additive and uniformly continuous and commutes with similarity transformations [1].

The utility of the Steiner point extends beyond its definition as a robust center of a set of static points. Locating the Steiner point of an object is helpful for many tasks, because Steiner point is an invariant point of an object, while a transform is used on it in certain ways, such as growing uniformly in all directions, moving in a line, and rotating around an axis [3]. By finding the Steiner point of an object, one can analyze some properties of an image [4]. To detect or recognize an object in an image, Steiner point can help us in some cases [5]. If two objects are similar but have different Steiner points, one can distinguish them in this way [6]. Tracking moving objects is now a popular approach for research workers [7–10]; if Steiner points of objects are referenced, they could save a large amount of computation.

In the early years, much work has been done on some algebraic and analytic structure and behavior of Steiner points, such as linear translation, continuity, and even affine translation of an object. Three important properties were studied and known as basic properties of Steiner points, which are shortly denoted by commutation, addition, and continuity [2, 11, 12]. Furthermore, the definition of Steiner point was generalized from a polytope to a nonempty compact subset *K* of ℝ^*n*^ [13]. Furthermore, the stability and eccentricity of Steiner point were researched and applied to mobile facility location [14]. To implement calculation of Steiner point, there are several alternatives [4]; one of the efficient ways refers to [1], which is based on the exterior angle of convex points in a polytope. In recent years, Steiner point has been extended to fuzzy set and provides an alternative strategy of defuzzication, which is regarded as the center of the fuzzy set [3, 15].

This approach focuses on implementing Steiner point of fuzzy set. The motivation is trying to find an efficient method to calculate the Steiner point of a fuzzy set. This paper is arranged as follows. In the second part, some definitions and properties of Steiner point of fuzzy set are investigated referring to the literatures. The third part discusses strategies to compute the Steiner point of a fuzzy set. Two main methods are proposed for calculating the Steiner point of a fuzzy set. In the fourth part, stability analysis of Steiner point of fuzzy set is proposed. In the fifth part, some experiments on image processing are presented. The last part of the paper contains the conclusions.

## 2. Definitions of Steiner Point and Properties

In the following let us suppose that *n*⩾0 is an integer. We denote by *𝒦* ^*n*^ the set of nonempty compact convex subsets of ℝ^*n*^. The set *𝒦* ^*n*^ is endowed with a linear structure in which the addition of two subsets and the multiplication of a subset by a positive real number are defined pointwise. We furthermore endow *𝒦* ^*n*^ with the Hausdorff metric *d*_*E*_. Let *S* ^*n*−1^ denote the unit sphere in ℝ^*n*^, and let *C*(*S* ^*n*−1^) denote the space of continuous functions from *S* ^*n*−1^ to ℝ, endowed with the supremum norm. Now, for *A* ∈ *𝒦* ^*n*^, we define the support function of *A*; see, for example, [16], by

(1) $$\begin{array}{c} h_{A}:S^{n-1}⟶\mathbb{R},\\ e⟼\max\left\{\langle a,e\rangle :a\in A\right\}, \end{array}$$

where 〈·, ·〉 denotes the usual inner product of ℝ^*n*^. The following definition is due to [3, 11].

**Definition 1**

The Steiner point of *A* in *K* ^*n*^ is defined as

(2) $$\begin{array}{c} S\left(A\right)=\frac{1}{V\left(B^{n}\right)}\int_{S^{n-1}}h_{A}\left(e\right)e\;d\lambda \left(e\right), \end{array}$$

where *e* ∈ *S* ^*n*−1^ varies over the unit vectors of ℝ^*n*^, *λ* is the Lebesgue measure on *S* ^*n*−1^, and *V*(*B* ^*n*^) is the volume of the unit ball *B* ^*n*^ of ℝ^*n*^. Notice that *s*(*A*) ∈ *A*.

We denote by *ℱ* ^*n*^ the set of all functions from [0,1] to *𝒦* ^*n*^ which are (i) decreasing and (ii) left continuous on (0,1] and continuous at 0. For a fuzzy set *u* ∈ *ℱ* ^*n*^ and a rigid motion *τ* we set *τu* : [0,1] → *K* ^*n*^, *α* ↦ *τ*(*u*(*α*)).

**Definition 2**

Let *μ* : [0,1]→[0,1] be a measure function. For *u* ∈ *ℱ* ^*n*^, let

(3) $$\begin{array}{c} S_{\mu }\left(u\right)=\int_{\left[0,1\right]}s\left(u\left(\alpha \right)\right)d\mu \left(\alpha \right), \end{array}$$

where *s* is the Steiner point of crisp sets. Then *S*_*μ*_ is a Steiner point of fuzzy set *u*.

Based on Definition 1, the following properties refer to [2, 12].

**Theorem 3**

Let *s*′ : *S* ^*n*−1^ → ℝ^*n*^ have the following properties:

(S1) for any *A*, *B* ∈ *K* ^*n*^, *s*′(*A* + *B*) = *s*′(*A*) + *s*′(*B*);
(S2) for *A* ∈ *K* ^*n*^ and any rigid motion *τ*, one has *s*′(*τA*) = *τs*′(*A*);
(S3) *s*′ is continuous.

Then *s*′ = *s* is the Steiner point. These three properties are described in [11] as addition, commutation, and continuity of Steiner point.

The following theorem is due to [3], which is an extension of Theorem 3 to the case of fuzzy set.

**Theorem 4**

A function *S* : *ℱ* ^*n*^ → *𝒦* ^*n*^ is called a Steiner point if it has the following properties:

(SF0) for any *u* ∈ *ℱ* ^*n*^, *S*(*u*) ∈ *u*(0);
(SF1) for any *u*, *v* ∈ *ℱ* ^*n*^, *S*(*u* + *v*) = *S*(*u*) + *S*(*v*);
(SF2) for *u* ∈ *𝒦* ^*n*^ and any rigid motion *τ*, one has *S*(*τu*) = *τS*(*u*);
(SF3) *S* is continuous.

As mentioned in [17, 18], a Steiner point of fuzzy set is not defined unambiguously by the properties (SF0)–(SF3). It is amazingly difficult to impose further properties on *S* to obtain uniqueness; it is an open question if this is possible in some reasonable way. For the purpose of calculating Steiner point of fuzzy set, we introduce the following definitions and lemmas [3].

**Definition 5**

Let *D* = (*α*_0_,…, *α*_*k*_) be a division of [0,1], which is 0 = *α*_0_ < *α*_1_ < ⋯<*α*_*k*_ = 1. Then one calls a fuzzy set *u* ∈ *ℱ* ^*n*^ a *D*-step fuzzy set if it is constant on [*α*_0_, *α*_1_], (*α*_1_, *α*_2_],…, (*α*_*k*−1_, *α*_*k*_], respectively. One denotes by *ℱ*_*D*_^*n*^ the set of all *D*-step fuzzy sets.

**Definition 6**

Let *D* = (*α*_0_,…, *α*_*k*_) be a division of  [0,1]. Let *s* : *ℱ*_*D*_^*n*^ → ℝ^*n*^ be a function fulfilling the properties (SF0)–(SF3) of Theorem 4. Then one calls a Steiner point *s*(*u*) ∈ ℝ^*n*^ a *D*-step Steiner point if it is constant on [*α*_0_, *α*_1_], (*α*_1_, *α*_2_],…, (*α*_*k*−1_, *α*_*k*_], respectively. One denotes by *𝕊*_*D*_^*n*^ the set of all *D*-step Steiner points.

**Lemma 7**

Let *D* = (*α*_0_,…, *α*_*k*_) be a division of [0,1]. Let *S* : *ℱ*_*D*_^*n*^ → ℝ^*n*^ be a function fulfilling the properties (SF0)–(SF3) of Theorem 4. Then there are unique real numbers *t*_1_,…, *t*_*k*_ such that *t*_1_ + ⋯+*t*_*k*_ = 1 and, for all *u* ∈ *ℱ*_*D*_^*n*^,

(4) $$\begin{array}{c} S\left(u\right)=t_{1}s\left(u\left(\alpha_{1}\right)\right)+\cdots +t_{k}s\left(u\left(\alpha_{k}\right)\right). \end{array}$$

**Lemma 8**

Let *D* = (*α*_0_,…, *α*_*k*_) be a division of [0,1]. Let *S* : *ℱ*_*D*_^*n*^ → ℝ^*n*^ be a function fulfilling the properties (SF0)–(SF3) of Theorem 4. Let *t*_1_,…, *t*_*k*_ be the unique real numbers fulfilling (4). Then *t*_1_,…, *t*_*k*_⩾0.

For the general case, the Steiner point of a fuzzy set can be calculated as follows.

**Lemma 9**

Let *S* : *ℱ*_*D*_^*n*^ → ℝ^*n*^ be a Steiner point. Then there is a measure function *μ* : [0,1]→[0,1] such that, for all *u* ∈ *ℱ* ^*n*^,

(5) $$\begin{array}{c} S\left(u\right)=\int_{\left[0,1\right]}s\left(u\left(\alpha \right)\right)d\mu \left(\alpha \right). \end{array}$$

## 3. Calculation of Steiner Point of Fuzzy Set

From Lemma 9, we know that computing Steiner point of a fuzzy set can be transformed to computing Steiner point of *α*-cut sets of a fuzzy set, which means that defuzzification is necessary in fuzzy set Steiner point computing. Defuzzification methods may be divided into two classes, considering either the horizontal or the vertical representation of the fuzzy set. In the former, one assigns to each possibility value a set of elements of the universe in agreement with that possibility value, that is, *α*-cut representation, while in the latter one assigns to each element of the universe a possibility value. In this paper we focus on the former and discuss a little about the later.

**Definition 10**

Let *α* ∈ [0,1] be a scale. Let *u* ∈ *ℱ* ^*n*^ be a fuzzy set. Then one calls an *α*-cut *u*(*α*) and *u*(*α*) = {*x* ∈ ℝ^*n*^ | *m*_*u*_(*x*) > *α*}, which is a crisp set.

Now there are two strategies to compute Steiner point of a fuzzy set. One is to find a series of *α*-cut sets of the fuzzy set, compute Steiner point for each *α*-cut set, and combine them in linear form as in (4). The other strategy is to try to find a crisp set in the *α*-cut sets, which has the same Steiner point with the fuzzy set. We will discuss these two methods in detail in the following.

In the case of step fuzzy set, we have fixed the number of *α*-cut sets, so it is easy to transform a fuzzy set into a series of crisp sets. But there may be a large number of *α*-cut sets, which are not necessary in calculating Seiner point of a fuzzy set. So, in this case, we prefer the step Steiner point, which is as defined in Definition 6.

From Lemma 7, it is known that once *k* *α*-cut sets of fuzzy set *u* are confirmed, which can be denoted by *u*(*α*_1_),…, *u*(*α*_*k*_), computing Steiner point of fuzzy set *u* is equivalent to computing Steiner points of the series of *k* *α*-cut sets, namely, *s*(*u*(*α*_1_)),…, *s*(*u*(*α*_*k*_)), and combining them by a series of weights *t*_1_,…, *t*_*k*_ which satisfy

(6) $$\begin{array}{c} t_{1}+\cdots +t_{k}=1 \end{array}$$

and *t*_1_⩾0,…, *t*_*k*_⩾0. One of the choices is simply taking

(7) $$\begin{array}{c} t_{i}=\alpha_{i}-\alpha_{i-1},\;\text{for}\;i=1,\ldots ,k, \end{array}$$

where *α*_0_ = 0. In the view of numerical computing, calculating Steiner point of a fuzzy set in this way can be much more complex, while a lot of level sets are taken for the fuzzy set.

For the sake of convenience in computing Steiner point of a fuzzy set, we introduce the second strategy (which is similarly approximate). Firstly, consider the following definition.

**Definition 11**

Let *u*(*α*_*o*_) be a *α*-cut of fuzzy set *u* ∈ *ℱ* ^*n*^. Then one calls *u*(*α*_*o*_) the optimal approximate crisp set to *u* if

(8) $$\begin{array}{c} d\left(u\left(\alpha_{o}\right),u\right)=\underset{\alpha \in [0,1]}{\min}d\left(u\left(\alpha \right),u\right), \end{array}$$

where *d*(*u*(*α*), *u*) is the distance between crisp set *u*(*α*) and fuzzy set *u*.

**Theorem 12**

Let *u*(*α*_*o*_), one of *α*-cut sets of fuzzy set *u*, be the optimal approximate crisp set to fuzzy set *u*. Then *S*(*u*(*α*_*o*_)), as defined in Definition 1, namely,

(9) $$\begin{array}{c} S\left(u\right)=S\left(u\left(\alpha_{o}\right)\right) \end{array}$$

is a Steiner point of fuzzy set *u*.

**Proof**

We prove that *S* satisfies (S1)–(S3) in Theorem 3. Denote by *τ* a real number and denote by *u*(*α*) and *v*(*α*) the *α*-cut sets of fuzzy sets *u*, *v* ∈ *ℱ* ^*n*^, respectively. According to [17], (*u* + *v*)(*α*) = *u*(*α*) + *v*(*α*) and (*τu*)(*α*) = *τu*(*α*); then

(10) $$\begin{array}{c} S\left(\left(u+v\right)\left(\alpha \right)\right)=S\left(u\left(\alpha \right)+v\left(\alpha \right)\right)=S\left(u\left(\alpha \right)\right)+S\left(v\left(\alpha \right)\right),\\ S\left(\tau u\left(\alpha \right)\right)=\tau S\left(u\left(\alpha \right)\right). \end{array}$$

So (S1) and (S2) are satisfied. Rather, more *S* is continuous, which is (S3). This completes the proof.

Another motivation is from [15, 17] and Lemma 9. If we rewrite (5) as

(11) $$\begin{array}{c} S\left(u\right)=\frac{\int_{\left[0,1\right]}s\left(u\left(\alpha \right)\right)d\mu \left(\alpha \right)}{\int_{\left[0,1\right]}d\mu \left(\alpha \right)}, \end{array}$$

it is clear that Steiner point of a fuzzy set is the average of the Steiner points of all the level sets *u*(*α*). Note that *s*(*u*(*α*)) is continuous with respect to *α*; by the Mean Value Theorem, there exists an $\overset{-}{\alpha }$ such that

(12) $$\begin{array}{c} S_{\mu }\left(u\right)=\overset{1}{\underset{0}{\int }}s\left(u\left(\alpha \right)\right)\mu \left(\alpha \right)d\alpha =s\left(u\left(\overset{-}{\alpha }\right)\right). \end{array}$$

**Theorem 13**

If *u* is a convex fuzzy set and *s*(*u*(*α*)) is continuous with respect to *α*, then $u(\overset{-}{\alpha })$ in (12) satisfies

(13) $$\begin{array}{c} d\left(u\left(\overset{-}{\alpha }\right),u\right)=\underset{\alpha \in \left[0,1\right]}{\min}d\left(u\left(\alpha \right),u\right). \end{array}$$

It follows from [15] that the Steiner point is a characteristic point of a fuzzy set in the sense of

(14) $$\begin{array}{c} \underset{x\in {\mathbb{R}}^{n}}{\inf}d\left(x,u\right)=d\left(s\left(u\right),u\right), \end{array}$$

where *d* is the *L*_2_-metric on fuzzy space *ℱ* ^*n*^. This implies the following.

**Theorem 14**

For any Steiner point of fuzzy set *u* ∈ *ℱ* ^*n*^ there is $s(u)\in u(\overset{-}{\alpha })$, where $u(\overset{-}{\alpha })=u(\alpha_{o})$ is an *α*-cut set of fuzzy set *u* and satisfies (8).

**Theorem 15**

A Steiner point of $u(\overset{-}{\alpha })$, which is an *α*-cut set of fuzzy set *u* and satisfies (8), is also a Steiner point of fuzzy set *u*; namely,

(15) $$\begin{array}{c} \underset{x\in u\left(\overset{-}{\alpha }\right)}{\inf}d\left(x,u\left(\overset{-}{\alpha }\right)\right)=d\left(s\left(u\left(\overset{-}{\alpha }\right)\right),u\left(\overset{-}{\alpha }\right)\right)=d\left(s\left(u\right),u\right). \end{array}$$

If it is reasonable, we can introduce the following definitions.

**Definition 16**

For $\tilde{A}\in {ℱ}^{2}$, one defines the support function of $\tilde{A}$ by

(16) $$\begin{array}{c} h_{\tilde{A}}:{ℱ}^{n-1}⟶\mathbb{R},\\ e⟼\max\left\{\langle \overset{-}{a},e\rangle :\overset{-}{a}=m_{\tilde{A}}\left(a\right)a,a\in A\right\}, \end{array}$$

where $m_{\tilde{A}}(a)$ is a membership function of the fuzzy set $\tilde{A}$, and

(17) A=A~(αo)={x∈A~(αo) ∣ d(A~(αo),A~)=min⁡α∈[0,1]d(A~(α),A~)}.

**Definition 17**

For $\tilde{A}\in {ℱ}^{n}$, the Steiner point of fuzzy set $\tilde{A}$ is given by

(18) $$\begin{array}{c} S\left(\tilde{A}\right)=\frac{1}{V\left(B^{n}\right)}\int_{S^{n-1}}h_{\tilde{A}}\left(e\right)e\;d\lambda \left(e\right), \end{array}$$

where *e* ∈ *S* ^*n*−1^ varies over the unit vectors of ℝ^*n*^, *λ* is the Lebesgue measure on *S* ^*n*−1^, and *V*(*B* ^*n*^) is the volume of the unit ball *B* ^*n*^ of ℝ^*n*^.

Now what we need is computing Steiner point based on crisp set, for a 2-dimensional case, based on polygon or convex polygon. For a 2D set, there are two steps to compute its Steiner point in numerical sense. The first step is to find all the convex points of the set and to form a convex polygon *A*, which is proved to be linear computational complex in [19]. The second step is referring to [2, 20]. Consider

(19) $$\begin{array}{c} S\left(A\right)=\overset{M}{\underset{i=1}{\sum }}\psi \left(p_{i},A\right)p_{i}, \end{array}$$

where *ψ*(*p*_*i*_, *A*), for *i* = 1,…, *M*, is the proportion to 2*π* of the external angle of convex polygon *A* at *p*_*i*_.

## 4. Stability Analysis of Steiner Point

From [15], we know the following fact. For *α* ∈ (0,1] any *α*-cut of a *u* ∈ *ℱ* ^*n*^ is a convex compact subset of ℝ^*n*^ and can be uniquely characterized by its so-called support function. Therefore, the fuzzy set itself is uniquely characterized by the function

(20) su(e,α) =sup⁡{〈e,a〉:a∈u(α),e∈Sn−1,α∈(0,1]}.

Now, the Steiner point *S*(*u*) of a fuzzy set *u* ∈ *ℱ* ^*n*^ with ||*u*||_1_ < *∞* is given by

(21) $$\begin{array}{c} S\left(u\right)=n\overset{1}{\underset{0}{\int }}\int_{S^{n-1}}s_{u}\left(e,\alpha \right)e\lambda \left(de\right)d\alpha , \end{array}$$

where *λ* is the Lebesgue measure on *S* ^*n*−1^ with *λ*(*S* ^*n*−1^) = 1. So if we denote

(22) $$\begin{array}{c} s\left(u\left(\alpha \right)\right)=n\int_{S^{n-1}}s_{u}\left(e,\alpha \right)e\lambda \left(de\right), \end{array}$$

the Steiner point *S*(*u*) of a fuzzy set *u* ∈ *ℱ* ^*n*^ takes the following form:

(23) $$\begin{array}{c} S\left(u\right)=\overset{1}{\underset{0}{\int }}s\left(u\left(\alpha \right)\right)d\alpha . \end{array}$$

Supposing *α*_1_, *α*_2_ ∈ (0,1] are any two values, the corresponding *α*-cut sets are *u*(*α*_1_) and *u*(*α*_2_). Now let us discuss their differences in computing the Steiner points:

(24) |S(u(α1))−S(u(α2))|=|n∫Sn−1su(e,α1)eλ(de) −n∫Sn−1su(e,α2)eλ(de)|⩽n∫Sn−1|su(e,α1)−su(e,α2)|eλ(de).

Considering that *s*_*u*_(*e*, *α*) is bounded, namely, there exists an upper boundary *K*(*e*) for each *e* ∈ *R* ^*n*−1^ such that

(25) $$\begin{array}{c} \left|s_{u}\left(e,\alpha_{1}\right)-s_{u}\left(e,\alpha_{2}\right)\right|⩽K\left(e\right)\left|\alpha_{1}-\alpha_{2}\right|. \end{array}$$

Therefore

(26) $$\begin{array}{c} \left|S\left(u\left(\alpha_{1}\right)\right)-S\left(u\left(\alpha_{2}\right)\right)\right|⩽K\left|\alpha_{1}-\alpha_{2}\right|, \end{array}$$

where

(27) $$\begin{array}{c} K=n\int_{S^{n-1}}K\left(e\right)e\lambda \left(de\right) \end{array}$$

is a Lipschitz constant.

Let us consider an example in the following. Suppose that *A* ∈ ℝ^*n*^ is a crisp set and $\tilde{A}\in {ℱ}^{n}$ is the corresponding fuzzy set with the following membership:

(28) $$\begin{array}{c} m_{\tilde{A}}\left(x\right)=\frac{1}{\sqrt{2\pi \sigma }}\;e^{-{\left(x-\mu \right)}^{2}/2\sigma^{2}}. \end{array}$$

That is, $\tilde{A}=\left\{\left(x,m_{\tilde{A}}\left(x\right)\right)\right\}$. The *α*-cut of fuzzy set $\tilde{A}$ is given by

(29) $$\begin{array}{c} {\tilde{A}}_{\alpha }=\left\{x\;|\;m_{\tilde{A}}\left(x\right)>\alpha \right\}=\left\{x\;|\;\frac{1}{\sqrt{2\pi \sigma }}e^{-{\left(x-\mu \right)}^{2}/2\sigma^{2}}>\alpha \right\}. \end{array}$$

Notice the following fact:

(30) $$\begin{array}{c} s_{\tilde{A}}\left(e,0\right)=\sup\left\{\langle e,x\rangle ,x\in {\tilde{A}}_{0}=A\right\}=h_{A}\left(e\right) \end{array}$$

and (25); then

(31) $$\begin{array}{c} \left|s_{\tilde{A}}\left(e,\alpha \right)-h_{A}\left(e\right)\right|=\left|s_{\tilde{A}}\left(e,\alpha \right)-s_{\tilde{A}}\left(e,0\right)\right|⩽K\left(e\right)\alpha . \end{array}$$

This leads to

(32) $$\begin{array}{c} \left|S\left({\tilde{A}}_{\alpha }\right)-S\left(A\right)\right|⩽K\alpha . \end{array}$$

## 5. Experimental Examples

**Figure 1:**
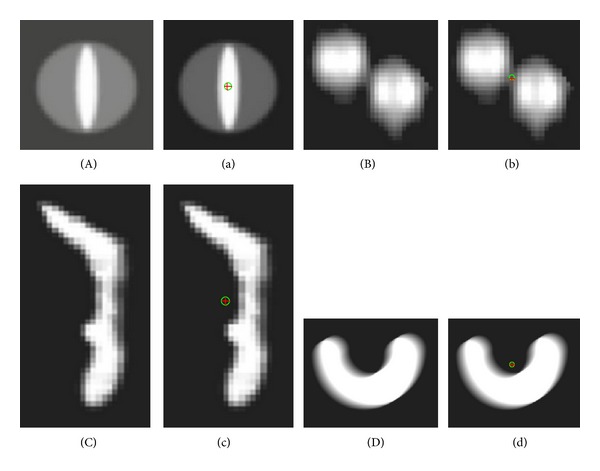
Test images used for evaluating two strategies of computing Steiner point of fuzzy set. The linear combination of Steiner point is marked by “o” and the approximate Steiner point is marked by “+.” (A) Synthetic fuzzy image. (a) The Steiner point of image (A). (B) A slice of a 3D MRA fuzzy segmented image of a human aorta. (b) The Steiner point of image (B). (C) Microscopy images of a born implant. (c) The Steiner point of image (C). (D) Synthetic fuzzy image. (d) The Steiner point of image (D).

In this section, we investigate four fuzzy images as an example, which were proposed in [21]; see Figure 1. Figure 1(A) shows a synthetic fuzzy set, described by a membership function radially nonincreasing from the centroid. Figure 1(B) presents a fuzzy segmented slice of a three-dimensional magnetic resonance angiography (MRA) image of a human aorta at the position where it splits into the two iliac arteries. Figure 1(C) shows a part of a histological light microscope image of a bone implant (inserted in a leg of a rabbit). The selected part of the fuzzy segmented image contains a bone area, surrounded by a nonbone area. Figure 1(D) shows a synthetic fuzzy set caused by motion, as described in [22], with len = 45 and theta = 30. We illustrate the implementation of the Steiner point of the fuzzy object in Figure 1 with the method presented in Section 3 and the comparison of the linear combination Steiner point with the proposed approximate Steiner point.

There are some technical issues that should be interpreted here. First, commonly not always all grays appear in an image, so we can find the minimum gray (denoted by *g*_min⁡_) and the maximum one (denoted by *g*_max⁡_) of the image by computing histogram of the image. Then, we divide the interval [*g*_min⁡_, *g*_max⁡_] into several levels with equal metric; that is, *g*_min⁡_ = *a*_0_, *a*_1_,…, *a*_*k*_ = *g*_max⁡_, and each gray corresponds to an *α*-cut of the fuzzy image. Second, in order to define the distance between the fuzzy image (the original image) and certain level image, we unify the grays of the fuzzy image by *J*_0_ = (*J* − *g*_min⁡_)/(*g*_max⁡_ − *g*_min⁡⁡_), where *J* is the gray matrix of the original fuzzy image, unify all the image grays of level *a*_*i*_, *i* = 0,…, *k*, which is denoted by *J*_*a*_, and compute the distance by ||*J*_0_ − *J*_*a*_||. Third, considering that the Steiner point of an object in an image is controlled by the shape of the object and the shape of the object depends greatly on boundary detecting, in most cases, boundaries of an object can be detected well in certain interval of gray, for example, in the middle part of interval [*g*_min⁡_, *g*_max⁡_]. So we can choose part of the interval [*g*_min⁡_, *g*_max⁡_] and divide it into several levels to compute the Steiner points. Here, we choose half of the interval [*g*_min⁡_, *g*_max⁡_] as the considered domain; namely, *G* = [*g*_min⁡_ + (1/4)(*g*_max⁡_ − *g*_min⁡_), *g*_max⁡_ − (1/4)(*g*_max⁡_ − *g*_min⁡_)] or *G* = (1/4)[3*g*_min⁡_ + *g*_max⁡_, 3*g*_max⁡_ − *g*_min⁡_].

In this paper, every degree of *α*-cut image is given according to the gray level of the image. Steiner points of the synthetic images, Figures 1(A) and 1(D), are shown in Figures 1(a) and 1(d), and Steiner points of the two real fuzzy segmented images, Figures 1(B) and 1(C), are given in Figures 1(b) and 1(c). In each image, the linear combination of Steiner point with equal weight of 1/8 for all levels is given and marked by “o.” Also, given in Figure 1, the approximate Steiner point is marked by “+,” which has the minimum distance between the fuzzy image and those gray images in all *α*-cut sets.

**Figure 2:**
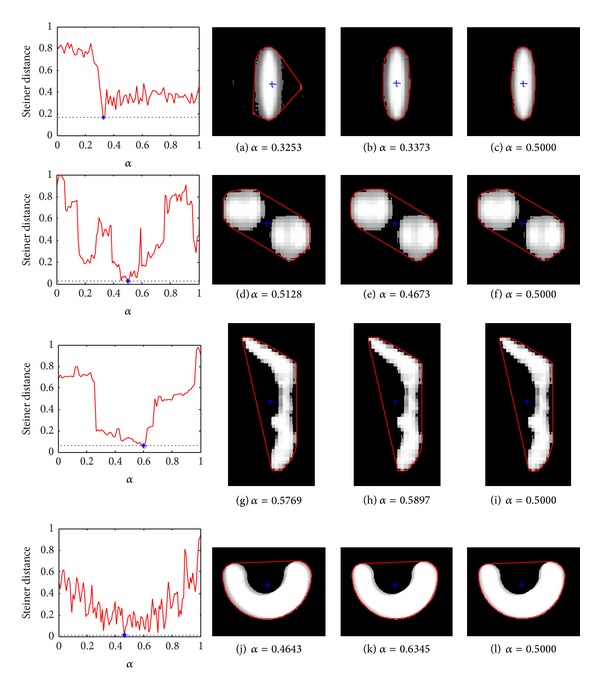
The Steiner point calculated by *α*-cut, and comparison of the distance between Steiner point of each gray level and the linear combined Steiner point. First column: plots of distances. The minimum is indicated with a star (*). Second column: the Steiner point calculated by *α*-cut at the minimum distance. Third column: the Steiner point calculated by *α*-cut at the second smallest distance. Fourth column: the Steiner point calculated by *α*-cut at *α* = 0.5.

In Figure 2, we plot the distance between the Steiner point of each gray level and the linear combined Steiner point in Figures 1(A), 1(B), 1(C), and 1(D). Also, we present their corresponding *α*-cut sets in Figure 1. The minimal value of distance between the approximate Steiner point and the combination of Steiner point is indicated by “*” in the plot. The corresponding value of *α* provides the optimal approximate *α*-cut. The three Steiner points calculated by *α*-cut set with the minimum distance, with the second smallest distance, and at *α* = 0.5, respectively, are shown the last three columns in Figure 1.  Table 1 presents the Steiner point of the object obtained by different methods and the distance between two Steiner points. var1 in Table 1 represents the variance of distance between the Steiner point of each gray level and the linear combination of Steiner point, while var2 presents the variance of distance between Steiner points of two adjacent gray levels. Results, expressed in var1 and var2, show that the distance between Steiner point of each gray level and the linear combination of Steiner point is more stable than the distance between Steiner points of two adjacent gray levels.

*Experiment Results Analysis*. Both two strategies of implementing Steiner point of fuzzy set have their own advantages and shortcuts. With the growing of the distance of image gray, the distance between the linear combined Steiner point and the Steiner points of each gray level do not enlarge rapidly. This means that the linear combined Steiner point shows more stability than the approximate Steiner point. Unfortunately, the former needs more computational time than the later, especially when more levels of gray are chosen to compute Steiner point. In our opinion, a suitable weight for combining the Steiner points of all the levels of fuzzy image is not reasonable to perform without having a particular application in mind. Also, from Figure 2, we see that the *α*-cut image is obtained by *α*-cut at optimal value of *α* in second column, which is chosen as the approximate Steiner point.

## 6. Conclusion

This approach focuses on implementing Steiner point of fuzzy set and some properties of Steiner point on fuzzy set. We try to find some efficient methods to compute Steiner point of fuzzy set. Two strategies of computing Steiner point of fuzzy set are proposed, namely, the linear combination of Steiner point, which calculates the Steiner point based on the approximate *α*-cut set. We also discuss some stable properties of Steiner point of fuzzy set and give some experiments on image processing. However, there are still some open problems which need more investigation while implementing Steiner point of a fuzzy set, such as how to choose each level of a fuzzy set, which is suitable for defuzzificating a fuzzy set? Does there exist unique choice of levels for fuzzy set in computing Steiner point? How to choose suitable weights for combining Steiner point of different levels? Those problems have high potential value in image processing.

## Figures and Tables

**Algorithm 1:**
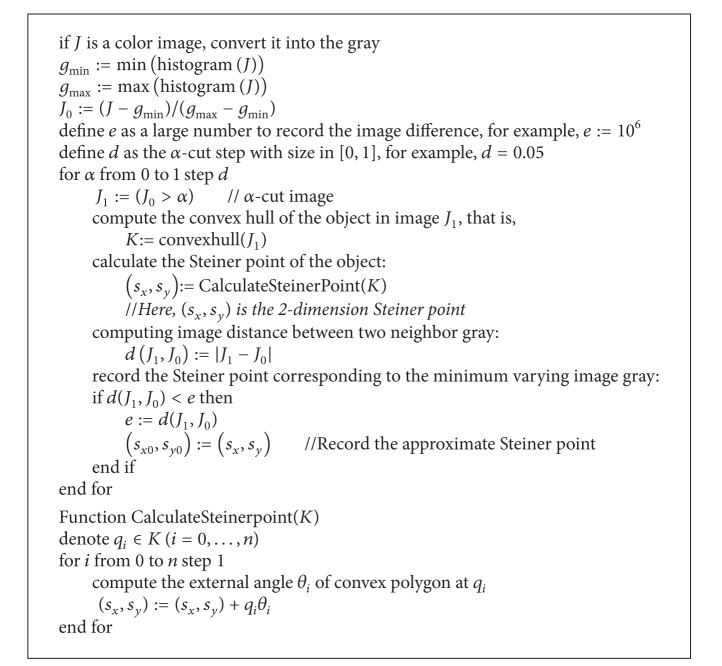
Implementations of algorithm.

**Table 1:** Comparison of the Steiner points obtained by two methods.

Figure	Approximate S.P.	D-step S.P.	Distance	var1	var2
Figure 1(A)	(83.49, 86.67)	(82.95, 86.42)	0.17	0.04	0.06
Figure 1(B)	(136.84, 123.30)	(136.27, 120.38)	0.03	0.08	0.05
Figure 1(C)	(64.61, 120.46)	(63.39, 120.06)	0.06	0.02	0.05
Figure 1(D)	(243.19, 165.84)	(242.26, 165.89)	0.01	0.04	0.04

## Appendix

### Implementations of Algorithm

See Algorithm 1.
